# Learning a novel technique to identify possible melanomas: are Australian general practitioners better than their U.K. colleagues?

**DOI:** 10.1186/1447-056X-8-3

**Published:** 2009-04-30

**Authors:** Tony Watson, Fiona M Walter, Annabel Wood, Helen Morris, Per Hall, Simone Karner, Jon Emery

**Affiliations:** 1 General Practice, School of Primary, Aboriginal and Rural Health Care, University of Western Australia, WA, Australia; 2 General Practice and Primary Care Research Unit, University of Cambridge, Cambridge, UK; 3 Addenbrookes Hospital, NHS Foundation Trust, Cambridge, UK

## Abstract

**Background:**

Spectrophotometric intracutaneous analysis (SIAscopy™) is a multispectral imaging technique that is used to identify 'suspicious' (i.e. potentially malignant) pigmented skin lesions for further investigation. The MoleMate™ system is a hand-held scanner that captures SIAscopy™ images that are then classified by the clinician using a computerized diagnostic algorithm designed for the primary health care setting. The objectives of this study were to test the effectiveness of a computer program designed to train health care workers to identify the diagnostic features of SIAscopy™ images and compare the results of a group of Australian and a group of English general practitioners (GPs).

**Methods:**

Thirty GPs recruited from the Perth (Western Australia) metropolitan area completed the training program at a workshop held in March 2008. The accuracy and speed of their pre- and post-test scores were then compared with those of a group of 18 GPs (including 10 GP registrars) who completed a similar program at two workshops held in Cambridge (U.K.) in March and April, 2007.

**Results:**

The median test score of the Australian GPs improved from 79.5% to 86.5% (median increase 5.5%; p < 0.001) while the median test score of the English GPs improved from 74.5% to 86.5% (median increase 9.5%; p < 0.001). The Australian GPs had significantly higher pre-test scores but there were no significant differences in post-test scores between the Australian and English GPs or between the GPs and GP registrars. There was no significant difference in scores between GPs with previous dermoscopy experience or dermatology training.

**Conclusion:**

Most of the SIAscopy™ features can be learnt to a reasonable degree of accuracy with this brief computer training program. Although the Australian GPs scored higher in the pre-test, both groups had similar levels of accuracy and speed in interpreting the SIAscopy™ features after completing the program. Scores were not affected by previous dermoscopy experience or dermatology training, which suggests that the MoleMate™ system is relatively easy to learn.

## Background

In Australia, skin cancer is the most common cancer, with melanoma being the fourth most common registrable cancer after prostate, colorectal, and breast cancer [1]. In 2003, there were 9,524 new cases of melanoma – a 14% increase in incidence since 1993 – and 1,146 deaths (764 males and 382 females) [1]. The risk of developing melanoma before the age of 75 is 1 in 24 for males and 1 in 34 for females [1]; melanoma is the most common cancer in the 20 to 39 year old age group [2].

Because the prognosis for melanoma is very good when lesions are excised 'early' (97.9% 10-year survival ≤ 0.75 mm Breslow thickness) and poor when they are not (40% 10-year survival > 4 mm Breslow thickness), the National Health and Medical Research Council has emphasized the importance of the early diagnosis of melanoma [3]. When compared with dermatologists, General Practitioners (GPs) can be highly sensitive but less specific for the diagnosis of melanoma [4]; this results in a relatively high proportion of excision biopsies [5] and secondary health care referrals [6] of benign pigmented skin lesions (PSLs).

To improve the accuracy of melanoma diagnosis by GPs, a variety of diagnostic algorithms and instruments have been developed. The most widely-published, evaluated and revised algorithms are the 'ABCD' [7] and 'Seven Point' [8] checklists, each of which has a significant sensitivity-specificity trade-off [9,10]. The most developed diagnostic instruments utilize dermoscopy, multispectral imaging, confocal laser microscopy, ultrasonography, optical coherence tomography, or magnetic resonance imaging [11]. Short training courses in dermoscopy, the cheapest and most-evaluated method, have been shown to increase the sensitivity of GPs for the diagnosis of melanoma without increasing their specificity [12,13]. A 2002 systematic review of dermoscopy concluded that "...dermoscopy improves the diagnostic accuracy for melanoma in comparison with inspection by the unaided eye, but only for experienced examiners" [14]. Clearly, to reduce the number (and cost) of biopsies and referrals of benign moles, GPs require more training and better tools to improve the specificity of their diagnosis of melanoma.

The MoleMate™ system incorporates a hand-held scanner that utilizes spectrophotometric intracutaneous analysis (SIAscopy™) to produce images of the light-absorbing chromophores haemoglobin, melanin and collagen in the epidermis and papillary dermis. Certain features of these images are combined with a customized diagnostic algorithm to predict the 'suspiciousness', or 'potential malignancy' of scanned lesions, indicating the need for biopsy or referral. Using an algorithm derived from patients referred to a hospital skin cancer clinic, SIAscopy™ has been shown to have a sensitivity of 82.7% (95% confidence interval [CI] 70.3% – 90.6%) and specificity of 80.1% (95% CI 75.1% – 84.2%) for the diagnosis of melanoma [15]. Receiver-operator characteristic analysis showed that the SIAscopy™ experts achieved a diagnostic accuracy similar to that of 11 dermatologists with 9 hours of dermoscopy training [16]. The MoleMate™ system, which uses a diagnostic algorithm derived from patients attending primary health care clinics, has been shown to have a sensitivity of 100% (95% CI 44% – 100%) and specificity of 78% (95% CI 75% – 82%) for the diagnosis of melanoma [17]. For comparison, a 2001 systematic review of studies of unaided clinical diagnostic accuracy for melanoma found that biopsy or referral sensitivity and specificity were 82–100% and 70–89% for dermatologists and 70–88% and 70–87% for Primary Care Physicians (GPs) [18]. The MoleMate™ system is currently undergoing a randomised controlled trial (RCT) in general practices in the east of England.

To facilitate the learning of the assessment of MoleMate™ scans by primary health care providers, a computer program, the MoleMate™ training program, was developed by the manufacturer, Astron Clinica™, and researchers from Cambridge University. The self-administered program takes approximately 90 minutes to complete and trains users to identify the typical SIAscopic™ features of pigmented skin lesions including seborrhoeic keratoses, haemangiomas, melanocytic naevi and melanomas.

In 2007, researchers from the University of Cambridge, UK, tested the MoleMate™ training program on 18 GPs [19]; the aim of this study was to test a similar group of Australian GPs and compare the results.

## Methods

### MoleMate^® ^images

**Figure 1:**
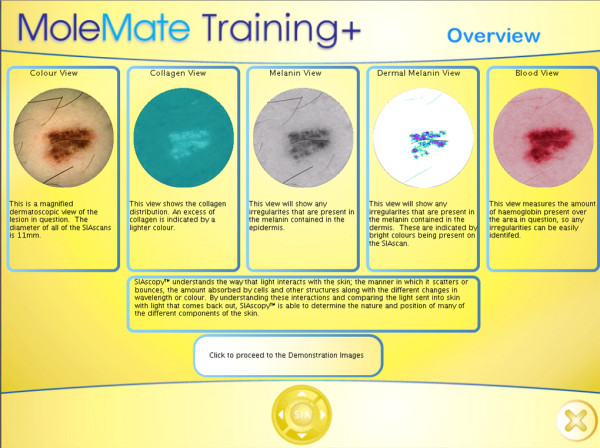
**Overview of MoleMate™ training program showing 'Views'**.

The computer program produces a colour dermatoscopic image and seven standard MoleMate™ images, or 'Views', to assess each lesion (see Figure 1 for examples of 'Views').

### MoleMate^® ^training program

**Figure 2:**
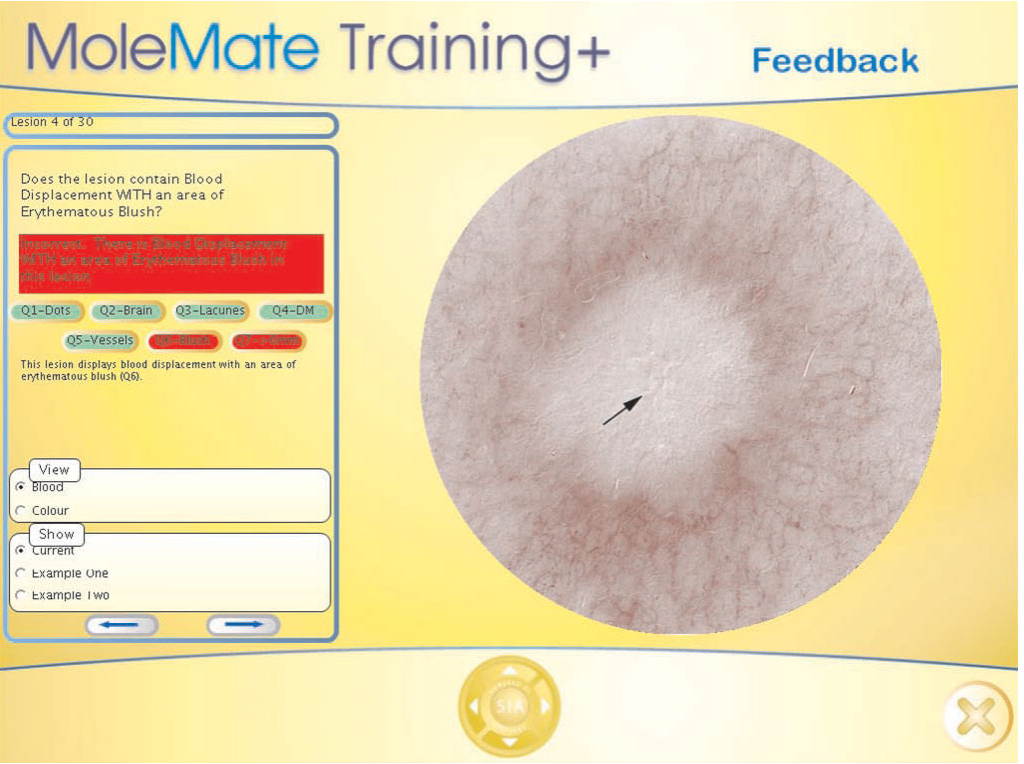
**Feedback section of MoleMate™ training program**.

The computer-based training program consists of four, self-administered sections:

**1. Demonstration **of the typical MoleMate™ features of 13 moles;

**2. Pre-test **of 30 MoleMate™ scans;

**3. Tailored feedback **of each pre-test answer for each scan (see Figure 2 for a feedback example);

**4. Post-test **of 30 MoleMate™ scans.

The program calculates a total score from the percentage of correct answers and the time it takes for each lesion to be assessed.

### Workshops

In Cambridge, 18 GPs were recruited by flyer and email to attend one of two evening workshops in Cambridge during March and April 2007. In Perth, 31 GPs were recruited by mail and email to attend an evening workshop in March 2008. After a 10-minute demonstration of the MoleMate™ system, the GPs were given 90 minutes, including a refreshment break, to complete the training program. One GP was unable to complete the program due to a computer malfunction.

### Analysis

Data were analysed using SPSS 15.0; pre- and post-test scores were compared using the Wilcoxon Signed-Rank Test for paired data and the Mann-Whitney U Test (using the exact two-tailed probability test) for unpaired data; p values less than 0.05 were regarded as statistically significant.

## Results

The personal data of the Australian and English GPs and GP registrars are summarized in Table 1.

**Table 1:** Summary of the personal data for GPs

	**Perth**	**Cambridge**	
	**GPs**	**GPs**	**GP registrars**
Average age (range) in years	51 (28–75)	48 (34–60)	31 (26–40)
Females	13 (43%)	4 (50%)	7 (70%)
Males	17 (57%)	4 (50%)	3 (30%)
Average years of general practice (range)	19 (2–45)	17 (9–25)	0.75 (0.5–1.0)
Routine use of dermoscopy	12 (40%)	n/a	n/a
Dermatology training	n/a	5 (63%)	1 (10%)

n/a Not assessed.

### Effectiveness of the MoleMate^® ^training program

#### Accuracy

The median pre- and post-test scores for the 30 Australian GPs were 79.5% (inter-quartile range (IQR 73.8% – 85.0%) and 86.5% (IQR 81.0% – 90.0%): median improvement 5.5% (IQR 1.0%–11.3%, p < 0.001).

The median pre- and post-test scores for the 18 English GPs were 74.5% (IQR 70.8% – 79.0%) and 86.5% (IQR 82.5% – 89.0%): median improvement 9.5% (IQR 6.5%–14.0%, p < 0.001).

**Figure 3:**
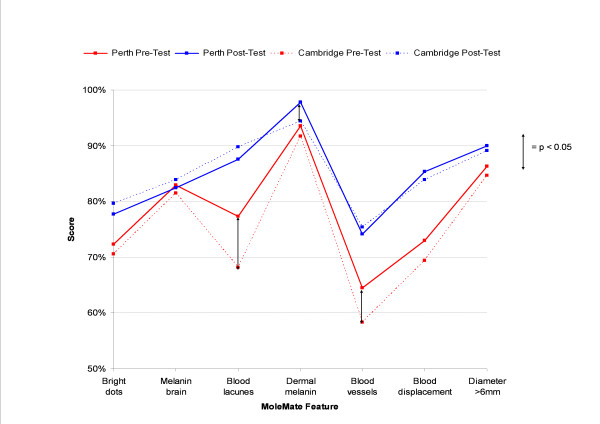
**Pre-test vs Post-test scores by MoleMate™ feature: Perth vs Cambridge GPs**.

The pre- and post-test scores for each MoleMate™ scan feature are shown in Figure 3. The Australian GPs significantly improved their scores for all the features except for the 'melanin brain' feature, while the English GPs significantly improved their scores for all the features except for the 'melanin brain' and 'dermal melanin' features.

The Australian GPs had higher pre-test scores than the English GPs (p = 0.045) but, except for a lower score for the 'dermal melanin' feature by the English GPs (p = 0.02), the post-test scores were not significantly different.

There were no significant differences between the total pre- or post-test scores of the 8 English GPs and 10 English GP registrars, but the registrars had significantly lower scores for the 'dermal melanin' feature on the pre-test (p = 0.036) and for the 'melanin brain' feature on the post-test (p = 0.016).

There were no significant differences in pre- or post-test scores between Australian GPs who routinely used dermoscopy and those who didn't and between English GPs with post-graduate dermatology training and those without.

#### Speed

The median average times taken by the Australian and English GPs to assess each lesion decreased from 43 to 35 seconds (p < 0.001) and from 47 to 36 seconds (p < 0.001), respectively.

There were no significant differences in the pre- or post-test times between the 30 Australian and 18 English GPs or between the 8 English GPs and 10 English GP registrars.

## Discussion

The most remarkable finding of this study was the similarity of the post-test scores of the Australian and English GPs; although English GPs scored significantly lower on the pre-test than the Australian GPs, their median post-test score was identical, with only one feature (dermal melanin) having a significantly lower score. Considering that the pre- and post-test skin lesions were different for both groups of GPs, and that the English GPs and GP registrars had similar post-test scores, the results suggest that the MoleMate™ training program is effective, (i.e. it improves the ability of individual GPs to assess SIAscopy™ features), and it is reliable (i.e. it is effective for different groups of GPs).

Presumably, the Australian GPs scored higher than the English GPs on the pre-test because they had more general practice experience and more experience diagnosing PSLs. Although 40% of the Australian GPs routinely used dermoscopy – some SIAscopy™ features are similar to those seen with dermoscopy – there were no significant differences in pre- or post-test scores between those who did and those who did not use dermoscopy. Similarly, there were no significant differences in pre- or post-test scores between the 33% of English GPs with postgraduate dermatology training and those without. This suggests that SIAscopy™ features are relatively easy to learn without previous dermatology experience.

Neither group improved their scores for the 'melanin brain' feature, which, except for the uniqueness of the image, is hard to explain; this feature is important for identifying seborrhoeic keratoses, benign lesions that are frequently referred to specialists.

GPs consistently scored greater than 90 percent for only one feature: dermal melanin. Further research is required to determine the amount of training GPs require to achieve the accuracy of a MoleMate™ expert.

There are some significant limitations to this study: the samples of GPs were small and not random, the English GPs were less experienced, and diagnostic accuracy was not evaluated. However, the training program was designed as an introduction to the use of the MoleMate™ system in clinical practice and should not be considered a stand-alone intervention. The training program and MoleMate™ system are currently being evaluated in an RCT in general practices in Cambridge, U.K.

Although the results of the SIAscopy™ feature testing cannot be extrapolated to the clinical domain, the results of the speed tests indicates that interpreting MoleMate™ scans would be relatively quick in clinical practice.

## Conclusion

Most of the SIAscopy™ features can be learnt to a reasonable degree of accuracy with this brief computer training program. Although the Australian GPs scored higher in the pre-test, both groups had similar levels of accuracy and speed in interpreting the SIAscopy™ features after completing the program. Scores were not affected by previous dermoscopy experience or dermatology training, indicating that the MoleMate™ system is relatively easy to learn without previous dermatology experience.

## Competing interests

TW and the workshops were funded by Astron Clinica™.

## Authors' contributions

JE, TW and SK conducted and analysed the Perth workshop. FW, PH, AW and HM conducted and analysed the Cambridge workshops. JE and FW conceived the study and helped draft the manuscript. TW performed the statistical analysis and drafted the manuscript. All authors read and approved the final manuscript.
